# Phase Transition of Single-Layer Molybdenum Disulfide Nanosheets under Mechanical Loading Based on Molecular Dynamics Simulations

**DOI:** 10.3390/ma11040502

**Published:** 2018-03-27

**Authors:** Haosheng Pang, Minglin Li, Chenghui Gao, Haili Huang, Weirong Zhuo, Jianyue Hu, Yaling Wan, Jing Luo, Weidong Wang

**Affiliations:** 1 School of Mechanical Engineering and Automation, Fuzhou University, Fuzhou 350108, China; m150210010@fzu.edu.cn (H.P.); n150220004@fzu.edu.cn (H.H.); m18065167496@163.com (W.Z.); n150220005@fzu.edu.cn (J.L.); 2 Fujian Key Laboratory of Medical Instrumentation and Pharmaceutical Technology, Fuzhou 350108, China; 3 Fujian Collaborative Innovation Center of High-End Manufacturing Equipment, Fuzhou 350108, China; 4 Fujian Province Special Equipment Inspection Institute, Fuzhou 35002, China; jianyuehu93@163.com; 5 BAK Power Battery Company, Shenzhen 518000, China; 18250160118@163.com; 6 School of Mechano-Electronic Engineering, Xidian University, Xi’an 710071, China; wangwd@mail.xidian.edu.cn

**Keywords:** MoS2, phase transition, molecular dynamics, nanoindentation, uniaxial compression

## Abstract

The single-layer molybdenum disulfide (SLMoS2) nanosheets have been experimentally discovered to exist in two different polymorphs, which exhibit different electrical properties, metallic or semiconducting. Herein, molecular dynamics (MD) simulations of nanoindentation and uniaxial compression were conducted to investigate the phase transition of SLMoS2 nanosheets. Typical load–deflection curves, stress–strain curves, and local atomic structures were obtained. The loading force decreases sharply and then increases again at a critical deflection under the nanoindentation, which is inferred to the phase transition. In addition to the layer thickness, some related bond lengths and bond angles were also found to suddenly change as the phase transition occurs. A bell-like hollow, so-called residual deformation, was found to form, mainly due to the lattice distortion around the waist of the bell. The effect of indenter size on the residual hollow was also analyzed. Under the uniaxial compression along the armchair direction, a different phase transition, a uniformly quadrilateral structure, was observed when the strain is greater than 27.7%. The quadrilateral structure was found to be stable and exhibit metallic conductivity in view of the first-principle calculation.

## 1. Introduction

Two-dimensional (2D) materials, constructed from one or a few layers of atoms, have attracted significant attention over the past decade since graphene was discovered by Geim in 2004 [1,2]. Among these, the single-layer molybdenum disulfide (SLMoS2), one of transition metal dichalcogenides (TMDs), has great promising potential for the applications of field-effect transistors (FETs) [3], phototransistors [4], flexible optoelectronic devices [5], and lithium-ion batteries (LIBs) [6] due to its unique electrical, optical, and mechanical properties. Different from single-atom-layer graphene, SLMoS2 is a structure of three atomic thickness with the transition metal (Mo) atomic layer sandwiched between two S atom layers [7]. It enables the SLMoS2 to exist in diverse polymorphs and consequently possesses different physical and chemical properties [8]. There are two phases of the typical SLMoS2, namely the 1T phase (octahedral) and 2H phase (trigonal prismatic) [9]. The 2H-phase MoS2 has the space group of P6/mmc and the semiconducting properties with a direct band gap [10]. The 1T phase MoS2 is metallic, hydrophilic, and metastable relative to the 2H-phase MoS2 [11].

In previous research, several methods have been demonstrated to induce the phase transition between the H and T phases in SLMoS2, including electron-beam irradiation [12], functionalization [13,14], intercalation [15,16], defect engineering [17,18], and chemical doping [19,20]. However, the studies on the phase transition for SLMoS2 induced by mechanical methods are quite limited. Wang et al. [21] recently investigated the plasticity resulting from the phase transition for the SLMoS2 film during nanoindentation simulations. It shows a sudden change in the S-S interlayer distance and S–Mo interlayer distance at critical deflection of indentation occurs, and a phase transition beneath the indenter is observed, which causes residual deformation. Similarly, Dang et al. [22] found the S-S intralayer distance of SLMoS2 decreases abruptly and a phase transition occurs prior to fracture of the membrane under uniaxial and multiaxial tension via molecular dynamics (MD) simulations. Li et al. [23] found the phase transition of SLMoS2 only occurs at a temperature of 1 K and at the moment of initial crack formation as tensed along the zigzag direction, and the new phase of quadrilateral structure remains stable after unloading. Zhao et al. [24] showed via MD simulations that the hexagonal structure of SLMoS2 can transform to a new quadrilateral structure under uniaxial tension along the zigzag direction for large deformations when the temperature range is from 4.2 to 40 K. The new phase remains stable after unloading and its Young’s modulus along the zigzag direction is about 2.5 times higher than that of the normal zigzag MoS2. The above mechanical methods have focused on the change in atomic structure of SLMoS2 in a small region under the indenter; however, they neglected the phase transition in the other residual region of the indentation. Besides, few scholars have studied the mechanical method of compression to induce the phase transition of SLMoS2.

In this work, MD simulations are performed to investigate the phase transition of SLMoS2 nanosheets under nanoindentation and uniaxial compression. The observed local atomic distortion and deformation phenomena of SLMoS2 nanosheets are analyzed and discussed. The bond lengths and bond angles related the atoms around the region of phase transition are measured. The stability and electrical properties of the obtained new phase are then evaluated by density functional theory (DFT) calculations.

## 2. Theoretical Models and Methods

The atomic structure of SLMoS2 is composed of a 2D honeycomb lattice that is occupied by a layer of Mo atoms, covalently sandwiched between the bottom layer of S atoms and the top layer of S atoms. A circular region with a radius of 100 Å, containing a total of 10,864 atoms, is defined extending from the center of the nanosheets for the nanoindentation MD simulations. Atoms outside the circular region are fixed as the boundary. A spherical diamond indenter with a radius of 10 Å is generated, which is made up of carbon atoms (total atoms of 749) and set as a rigid body. The initial distance between the bottom S layer and the top S layer is about 3.24 Å. The spherical indenter is loaded along the *z*-axis. Under the uniaxial compression, rectangular SLMoS2 nanosheets are conducted, and the periodic boundary conditions are applied in the basal plane. The compressed strain is applied along the *x*-axis (the armchair direction). The indentation rate and the strain rate were set to 0.2 Å/ps and 10^−5^ ps^−1^, respectively, referring to our previous work [21,23].

All simulations were performed with open source code, i.e., Large-Scale Atomic/Molecular Massively Parallel Simulator (LAMMPS). The interaction between carbon atoms in the diamond indenter was determined via the adaptive intermolecular reactive empirical bond-order (AIREBO) potential, which has predicted the mechanical properties of carbon structures in the previous study [21,25]. The atomic interactions in SLMoS2 were determined via the reactive empirical bond-order (REBO) potentials, which has recently been demonstrated to be more accurate in describing the elastic properties of SLMoS2 than the other interatomic potentials such as Stillinger–Weber (SW) and the consistent valence force field (CVFF) [26] and has been used to efficiently calculate the breaking force of SLMoS2 under uniaxial tension [23,27]. The interaction between the SLMoS2 and the diamond indenter was determined via the Lennard–Jones (LJ) potential, which has been verified in our previous works [21]. Before the nanoindentation and compression process, the energy of the system was minimized by the conjugated gradient method, and the isothermal–isobaric (NPT) ensemble controlled by the Nosé-Hoover method was then employed for system relaxation at a temperature of 0.1 K and a pressure of 0.1 bar (the time step is set to 1 fs). With an ambient temperature of 0.1 K, the interference of thermal fluctuation to the simulation could be maximally eliminated, and the intrinsic phase transition of SLMoS2 was thus expected to be revealed.

A periodic cell of SLMoS2, consisting of 24 atoms, was used in DFT calculations. Along with the thickness direction of SLMoS2, a vacuum layer of 20 Å is added to the cell. The calculations were performed using the CASTEP code, which is based on DFT and the plane-wave pseudopotential method. The PBE exchange–correlation function, which is adopted in the generalized gradient approximation (GGA), is used to solve the electronic system. After verification and reference, a 9 × 9 × 1 grid for the k-point and an energy cut-off of 400 eV were consistently used in our calculations. The energy tolerance was set to 2.0 × 10^−6^ eV.

The Open Visualization Tool (OVITO) was used to visualize the atomic structures obtained from MD simulations.

## 3. Results and Discussions

### 3.1. Nanoindentation of SLMoS2 Nanosheets

**Figure 1:**
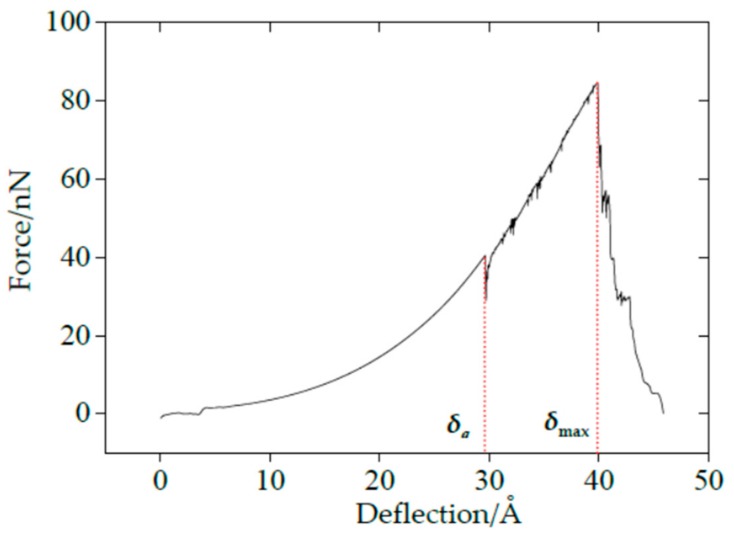
The force–deflection curve of SLMoS2 nanosheets.

The force–deflection curves under the nanoindentation for SLMoS2 nanosheets were obtained from the indenter load (force) and the indentation depth (deflection) calculated via MD simulations. A typical force–deflection curve is shown in Figure 1. The applied force suddenly drops to 0 nN once the deflection exceeds 40.14 Å, in which the corresponding deformation is defined as the maximum critical deflection (*δ_max_*). Different from the monotonic increase of loading force in simulations of nanoindentation for single-layer graphene films [28], there is another sudden decrease in loading force at the deflection of 29.72 Å, in which the deformation is defined as the first critical deflection (*δ_a_*). The sudden decrease also appears in the curve between the total system energy and the deflection, which is not shown here [21].

**Figure 2:**
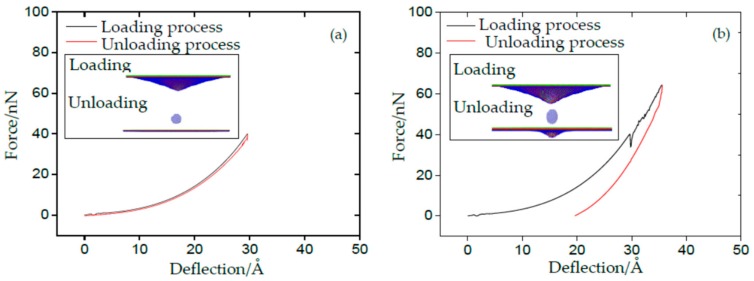
The force–deflection curves for the loading–unloading–reloading process of SLMoS2 nanosheets: (**a**) The deflection is smaller than *δ_a_*; (**b**) The deflection is greater than the *δ_a_* but smaller than *δ_max_*.

According to the theory of the properties of metals and alloys [29], when the external force is removed, the deformation of materials disappears and completely restores to its original shape, known as elasticity. On the contrary, the plasticity is defined as the permanent and unrecoverable deformation of materials after unloading. Compared with the yield process in the uniaxial tensile test of metal materials, the sudden drop in the force–deflection curve of SLMoS2 nanosheets can be attributed to lattice distortions, then resulting in plastic deformation. Therefore, we conducted a serial of loading and unloading simulations under nanoindentation. As for the loading process, the indenter moved down to penetrate the SLMoS2 nanosheets. The force–deflection curve is identical to the one shown in Figure 1, with insignificant difference. As for the first unloading process, the indenter moved back before the deflection reached *δ_a_*, as shown in Figure 2a. Obviously, the unloading curve is coincident with the loading curve, and the deformed SLMoS2 nanosheets can recover to their initial states (see the inset of Figure 2a). Perfect elastic deformation is appeared, as the deflection is smaller than the first critical deflection under nanoindentation. Another unloading process was conducted by moving the indenter back when the initial deflection was greater than *δ_a_* but less than *δ_max_*, as seen in Figure 2b. A residual pit can be observed in the inset of Figure 2b, which inferred that the plastic deformation occurred.

**Figure 3:**
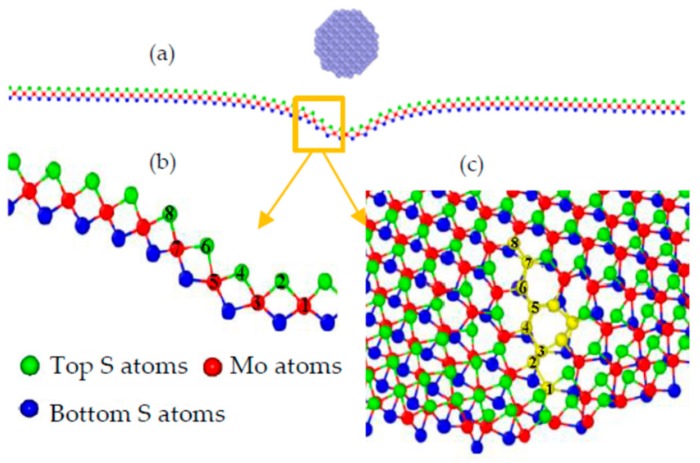
The atomic configuration at the cross section of the deformed SLMoS2 (**a**); the labeled atoms in the distorted area (**b**); the zoom in view of the residual hollow (**c**).

The residual bell-like hollow was characterized for an improved understanding of the intrinsic plastic deformation. Figure 3a shows the atomic configuration at the cross section, and a small region beneath the indenter was subjected to obviously unrecoverable distortion. When the distorted lattice is zoomed in, as shown in Figure 3b, the atom right under the indenter can be labeled as 1, and its surrounding atoms along the cross section labeled 2, 3, …, 8. It is found that the lattice disorder around the waist of the residual bell, which is close to Atom 4 in Figure 3c, can be substantially worse than that around the bottom of the bell. This situation can be clearly illustrated by accessing the evolutions of bond lengths and bond angles.

**Figure 4:**
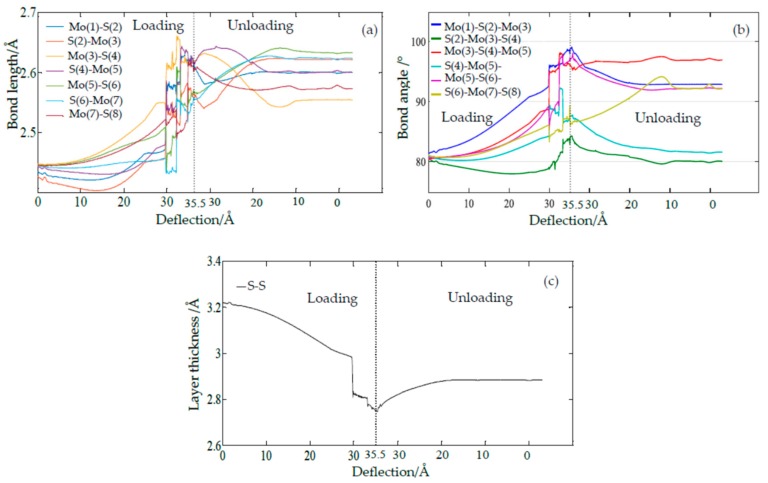
The S–Mo bond lengths (**a**), S–Mo–S bond angles and Mo–S–Mo angles (**b**), and the layer thickness of S–S (**c**) versus deflection during the loading process and unloading process (the labeled atoms are shown in Figure 3).

**Figure 5:**
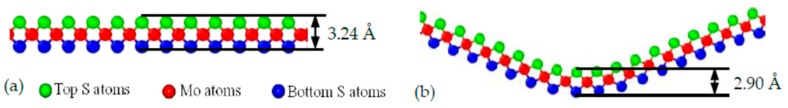
The atomic structures of SLMoS2 nanosheets in the process of nanoindentation: (**a**) before loading; (**b**) after unloading.

In the previous work [21], the plastic deformation was mainly attributed to the sudden shrink in the layer thickness. However, when we monitored the bond lengths and bond angles of those labeled atoms during the loading and unloading processes, it was found that every bond length and bond angle varies smoothly before the deflection reaches *δ_a_*, and then suddenly changes upward or downward until it returns to a stable terminal condition, as seen in Figure 4a,b. The order of their contribution to the residual deformation can be evaluated by their deviations from their initial bond lengths. The maximum contribution of the bond length is found to be the bond of Mo(5)–S(6) for its deviations of 7.69%, whereas the maximum contribution of bond angle is granted to the Mo(3)–S(4)–Mo(5) for its deviations of 20.07%. It can be seen that the maximum contributions of the bond lengths and bond angles result from the 3th, 4th, 5th, and 6th atoms, which are around the waist of the bell. Here, we also display the evolution of the layer thickness, as shown in Figure 4c. The layer thickness is defined as the distance between the top and the bottom S layers, as shown in Figure 5. Herein, the vertical distance between the top S atom (labeled 2 in Figure 3b) and its relative bottom S atom right underneath the indenter are measured and plotted to show the evolution of the layer thickness during the indentation. The layer thickness is found to suddenly decrease during the loading process and then gradually increases to a stable value, with the deviations of 10.49% after the unloading process. Though significant residual deformation of the layer thickness was observed in this paper and our previous work [21], the bond length and bond angle of the labeled atoms around the waist of the bell also have considerable residual deformation. The maximum deviation is attributed to the bond angle (20.07%), which is much greater than that of the layer thickness (10.49%). Combining the atomic structure and the evolutions of the bond lengths and bond angles, it can be inferred that the lattice distortion around the waist of the bell is substantially worse than that around the bottom of the bell.

### 3.2. Effect of Indenter Size on the Plastic Deformation

**Figure 6:**
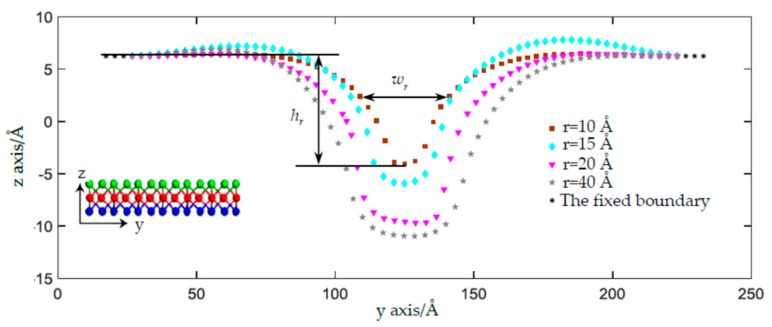
The sectional view of atoms in residual indentation with different indenter radii of 10 Å, 15 Å, 20 Å, and 40 Å at the deflection exceeding *δ_a_* after the unloading process. The rectangular, prismatic, triangular, and pentagonal points are the atomic positions of the Mo atoms close to the plane (*yz* plane) crossing the center of the indenter.

As shown in Figure 3a, the shape and size of the residual deformation under the nanoindentation were found to be close to those of the indenter. However, if the radius of the indenter increases, how does the residual deformation respond? To investigate the effect of the indenter on the residual deformation, we increased the indenter radius *r* and extracted the atomic configuration of the middle Mo layer at the symmetric crossing section plane, i.e., the *yz* plane, as shown in Figure 6. We defined three factors to characterize the residual deformation under different indenter radii: (1) the depth of the residual deformed bell, *h_r_*, i.e., the distance between the fixed Mo atoms and the lowest Mo atom, (2) the width of the residual bell, *w_r_*, i.e., the distance between the two Mo atoms around the half depth of the bell, and (3) the ratio of *r_d_* = *w_r_*/*h_r_*. The greater the values of *h_r_*, *w_r_*, and *r_d_* are, the much more substantial the residual deformation will be. Increasing the indenter size from 10 Å to 15 Å, to 20 Å, and to 40 Å, the deformations become more substantial as the depth of the residual bell *h_r_* increases from 10.29 Å to 12.18 Å, to 15.93 Å, and to 17.19 Å, the width of the residual bell *w_r_* increases from 20.5 Å, to 27.0 Å, to 33.6 Å, and to 44.9 Å, and the ratios of *r_d_* become 2.0, 2.2, 2.1, and 2.6. It is worth noting that a saddle-shape bulge occurs at the edge of the 15 Å indenter, which has the possibility of reducing its *h_r_* and *w_r_*. Moreover, when the radius of the indenter increases to more than 20 Å, the ratio between the radius of the SLMoS2 sheet and the indenter radius will become smaller than 5, which indicates that the indenter cannot be regarded as a point loading and will decrease the precision and accuracy of the MD simulation. When we looked into the evolutions of bond lengths and bond angles around the distorted lattices (given in the supplementary materials), it was found that the variation trends of layer thickness, every bond length, and every bond angle are the same as those shown in Figure 4. The above results again demonstrate that the phase transitions result not only from the sudden reduction in layer thickness but also from the distorted lattice around the waist of the bell.

### 3.3. Uniaxial Compression of SLMoS2 Nanosheets

**Figure 7:**
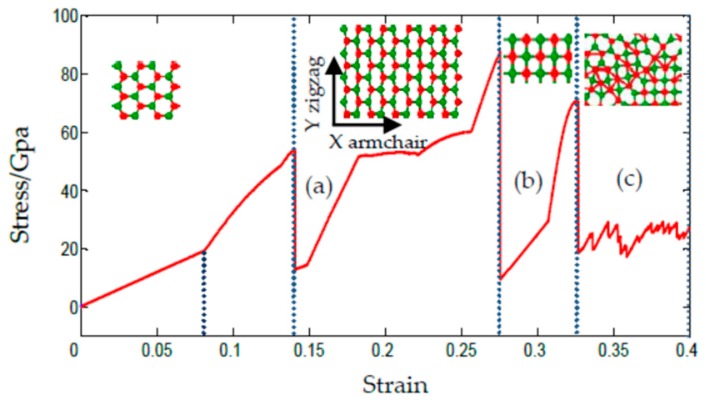
The stress–strain curves of the SLMoS2 nanosheets under uniaxial compression along the armchair direction. The insets show (**a**) The distorted hexagonal lattice; (**b**) the quadrilateral lattice; (**c**) the buckling.

The phase transition under the uniaxial compression of the SLMoS2 nanosheets was also investigated. A series of SLMoS2 nanosheets with different sizes and aspect ratios were compressed along the armchair direction, and only specimens that had an aspect ratio less than 0.85 and a size of less than 40 Å encountered a substantial phase transition. A typical sample with a size of 34.99 × 41.16 Å^2^ is shown in Figure 7, where a new quadrilateral lattice occurs at a compressive strain of 27.7% (Figure 7b). Before the quadrilateral lattice, the initial hexagonal lattice gradually becomes prolate, as shown in Figure 7a. New bonds are formed by the localized Mo–Mo atoms when the compressive strain is about 32.7%, as seen in Figure 7c. Note that there are several folds in the stress–strain curve, such as the points of 8.2%, 15%, 18.5%, and so on, which means that a slightly distorted bond length and bond angle are present. The quadrilateral lattice is then found to be stable when subjected to the energy minimization and then the tension. It is worth noting that, unlike [24], the ambient temperature in our simulations was set to 0.1 K. In [24], they focused their studies on the effect of temperature on the mechanical properties of SLMoS2 and found that a phase transition under the uniaxial compression occurred at below 40 K, e.g., 4.2 K. Herein, we focused our simulations on the phase transition of SLMoS2 under compression with a temperature of 0.1 K, which was expected to maximally eliminate the interference of thermal fluctuation and reveal the intrinsic phase transition of SLMoS2. It is also worth noting that the new quadrilateral phase occurs only when the compressive strain is greater than 27.7% and that the ideal limit of compressive strain of SLMoS2 is higher than 30%, and this is basically owed to the small size of the model, i.e., a size of less than 40 Å. Currently, such simulation results are useless for practical applications. However, to the best of our knowledge, by carefully preparing a sample of one-dimensional Si nanowires and precisely controlling the experimental procedure, one can obtain a higher elastic strain that approaches the theoretical elastic limit of silicon nanowires [30].

**Figure 8:**
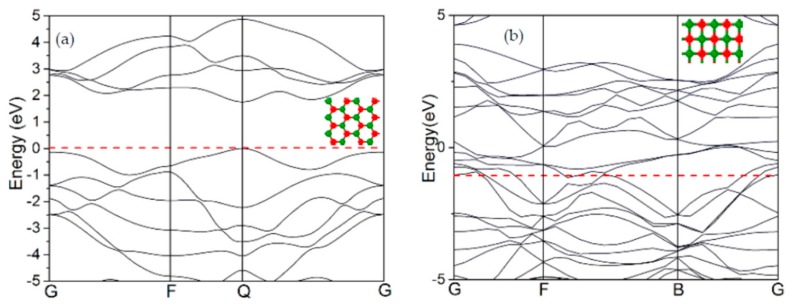
The band structures of (**a**) the initial hexagonal SLMoS2 nanosheets (semiconducting) and (**b**) the new phase of the quadrilateral structure (metallic).

The band structure of the new quadrilateral lattice was calculated via density functional theory (DFT) and was compared with the initial SLMoS2 nanosheets. Figure 8a shows the band structure of the initial hexagonal lattices, which show semiconducting nanosheets with a direct band gap of around 1.8 eV. However, there is no band gap around the Fermi energy level (red dashed line) in the quadrilateral lattice, shown in Figure 8b, which indicates a metallic property.

## 4. Conclusions

In summary, phase transitions of the SLMoS2 nanosheets under nanoindentation and uniaxial compression were investigated via MD simulations. Loading and unloading processes were conducted to investigate the atomic structural deformation with different initial loading conditions. Our results reveal the underlying mechanism of residual deformation under nanoindentation, and a new phase transition under uniaxial compression was discovered. The new quadrilateral lattice was found to be metallic, which paves the way to the design of a new heterogeneous composite that consists of both a quadrilateral lattice and a hexagonal lattice. In other words, based on the results in this paper, metallic and semiconducting materials can be theoretically combined in SLMoS2 nanosheets under different local strains in the future.

This work can provide a new perspective on the mechanism of the phase transition for the SLMoS2 nanosheet via strain engineering, which is helpful for its engineering applications in the design of devices and new material structures.

## Supplementary Materials

The following are available online at http://www.mdpi.com/1996-1944/11/4/502/s1, Figure S1: The Mo-S and S-Mo bond lengths (**a**, **d** and **h**), S-Mo-S bond angles and Mo-S-Mo angles (**b**, **e** and **i**), and layer thickness of S-S (**c**, **f** and **j**) versus deflection during the loading process and unloading process with indenter radii of 15 Å, 20 Å and 40 Å (The labeled atoms are shown in Figure 3).
