# Supplementary material for: Phase Transition of Single-Layer Molybdenum Disulfide Nanosheets under Mechanical Loading Based on Molecular Dynamics Simulations
Source: Materials (Basel). 2018 Mar 27;11(4):502. doi: 10.3390/ma11040502 (PMC5951348; doi:10.3390/ma11040502)
Supplement: Supplementary file 1 [file materials-11-00502-s001.pdf]

materials

Supplementary

Phase Transition of Single-Layer Molybdenum Disulfide Nanosheets under Mechanical Loading Based on Molecular Dynamics Simulations

Haosheng Pang [1], Minglin Li [1,2,3]*, Chenghui Gao [1,3]*, Haili Huang [1], Weirong Zhuo [1], Jianyue Hu [4], Yaling Wan [5], Jing Luo [1] and Weidong Wang [6]

[1] School of Mechanical Engineering and Automation, Fuzhou University, Fuzhou 350108, China; m150210010@fzu.edu.cn (H.P.); n150220004@fzu.edu.cn (H.H.); m18065167496@163.com (W.Z.); n150220005@fzu.edu.cn (J.L.)

[2] Fujian key laboratory of medical instrumentation and pharmaceutical technology, Fuzhou 350108, China

[3] Fujian collaborative innovation center of high-end manufacturing equipment, Fuzhou 350108, China

[4] Fujian Province Special Equipment Inspection Institute, Fuzhou 35002, China; jianyuehu93@163.com (J.H.)

[5] BAK Power Battery Company, Shenzhen 518000, China; 18250160118@163.com (Y.W)

[6] School of Mechano-Electronic Engineering, Xidian University, Xi'an 710071, China; wangwd@mail.xidian.edu.cn (W.W.)

* Correspondence: liminglin@fzu.edu.cn (M.L.); gch@fzu.edu.cn (C.G.)

Received: 11 February 2018; Accepted: 22 March 2018; Published: 26 March 2018

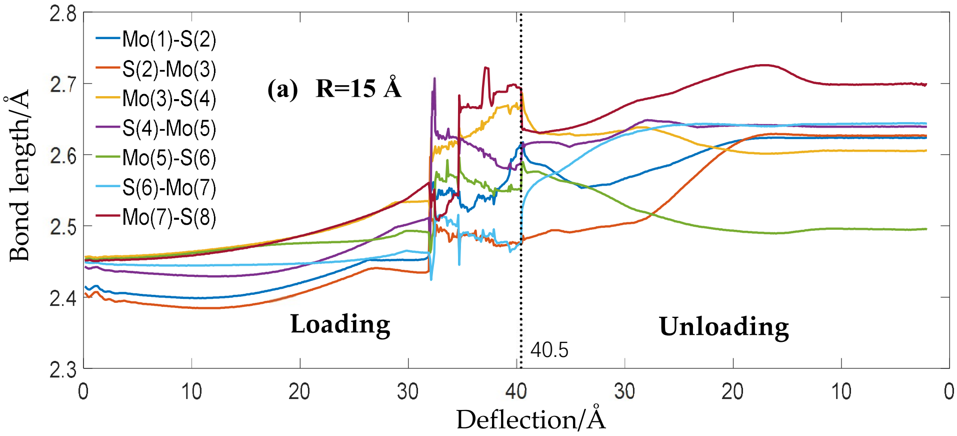

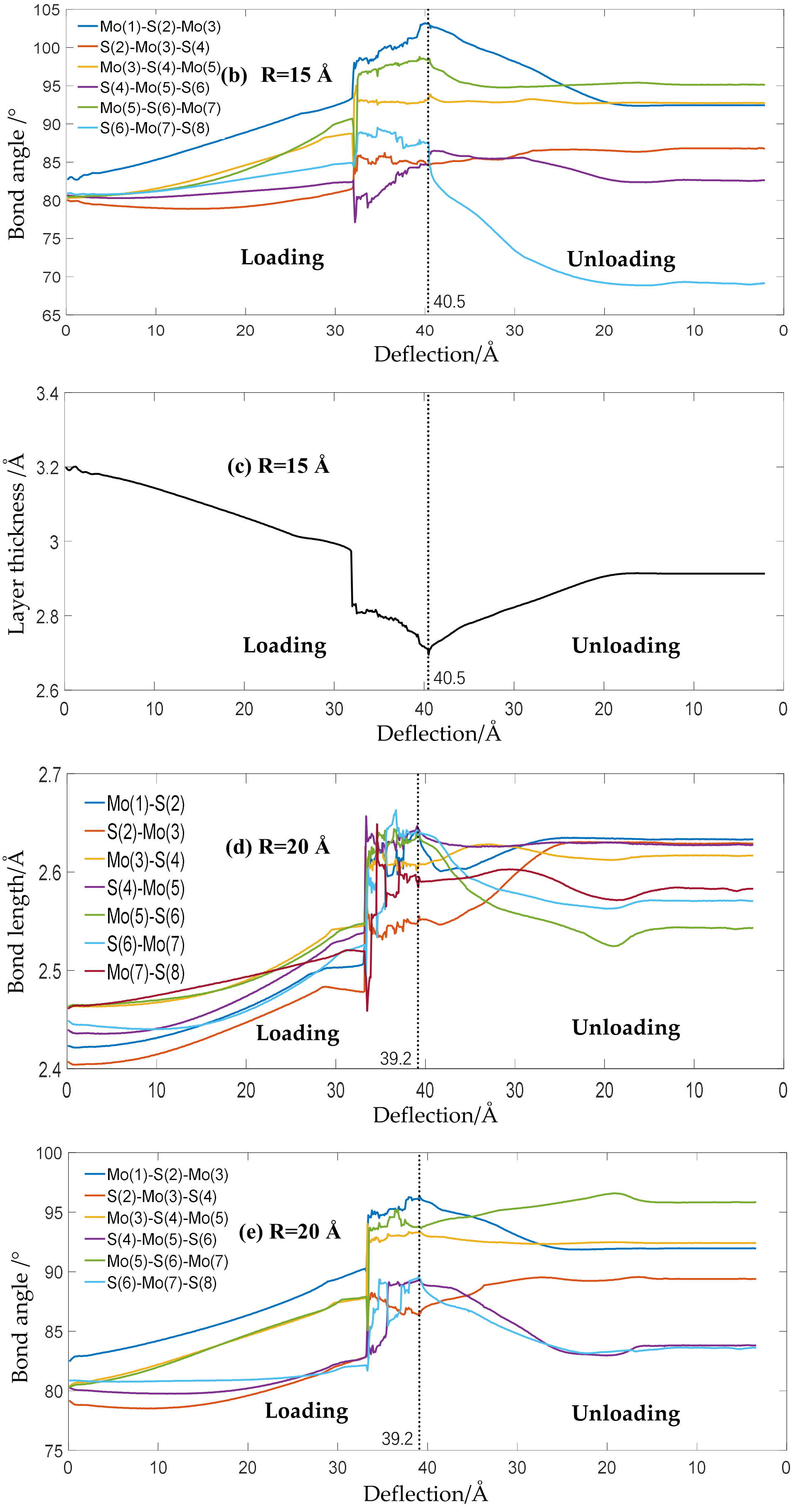

(b) R=15 Å
Mo(1)-S(2)-Mo(3)
S(2)-Mo(3)-S(4)
Mo(3)-S(4)-Mo(5)
S(4)-Mo(5)-S(6)
Mo(5)-S(6)-Mo(7)
S(6)-Mo(7)-S(8)
Bond angle /°
Loading
Unloading
40.5
Deflection/Å
(c) R=15 Å
Layer thickness /Å
Loading
Unloading
40.5
Deflection/Å
(d) R=20 Å
Mo(1)-S(2)
S(2)-Mo(3)
Mo(3)-S(4)
S(4)-Mo(5)
Mo(5)-S(6)
S(6)-Mo(7)
Mo(7)-S(8)
Bond length/Å
Loading
Unloading
39.2
Deflection/Å
(e) R=20 Å
Mo(1)-S(2)-Mo(3)
S(2)-Mo(3)-S(4)
Mo(3)-S(4)-Mo(5)
S(4)-Mo(5)-S(6)
Mo(5)-S(6)-Mo(7)
S(6)-Mo(7)-S(8)
Bond angle /°
Loading
Unloading
39.2
Deflection/Å

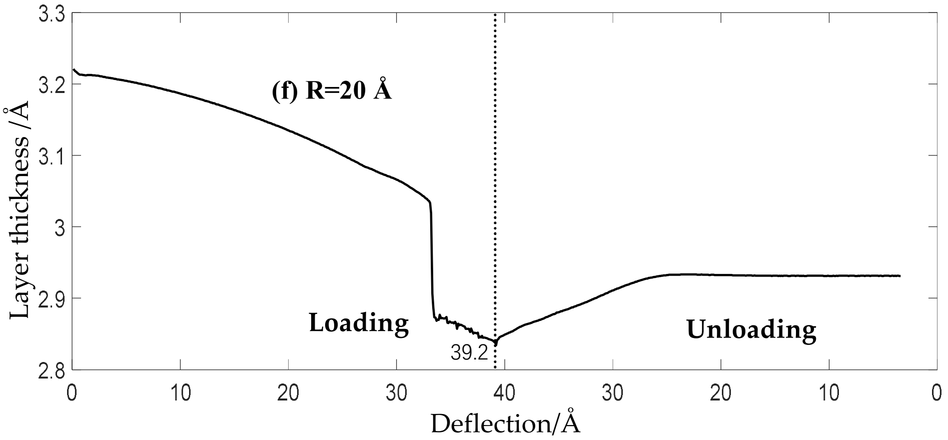
(f) R=20 Å
Layer thickness /Å
Deflection/Å
Loading
Unloading
39.2
3.3
3.2
3.1
3
2.9
2.8
0
10
20
30
40
30
20
10
0

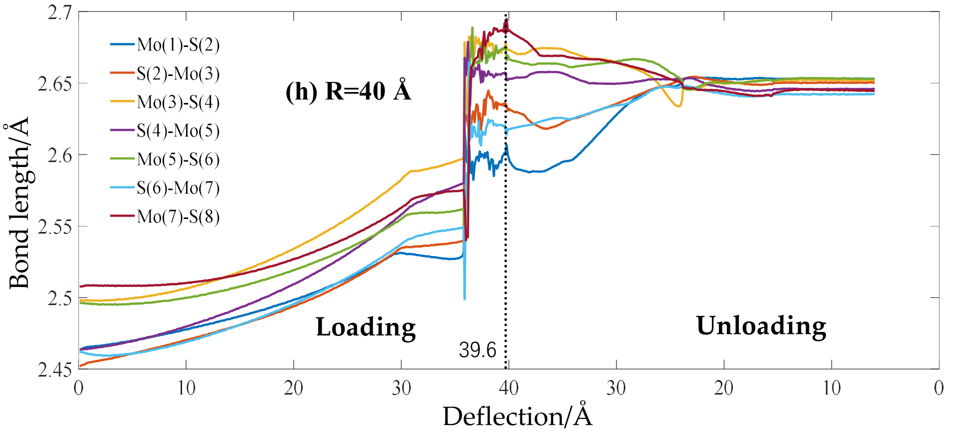
(h) R=40 Å
Mo(1)-S(2)
S(2)-Mo(3)
Mo(3)-S(4)
S(4)-Mo(5)
Mo(5)-S(6)
S(6)-Mo(7)
Mo(7)-S(8)
Bond length/Å
Deflection/Å
Loading
Unloading
39.6
2.7
2.65
2.6
2.55
2.5
2.45
0
10
20
30
40
30
20
10
0

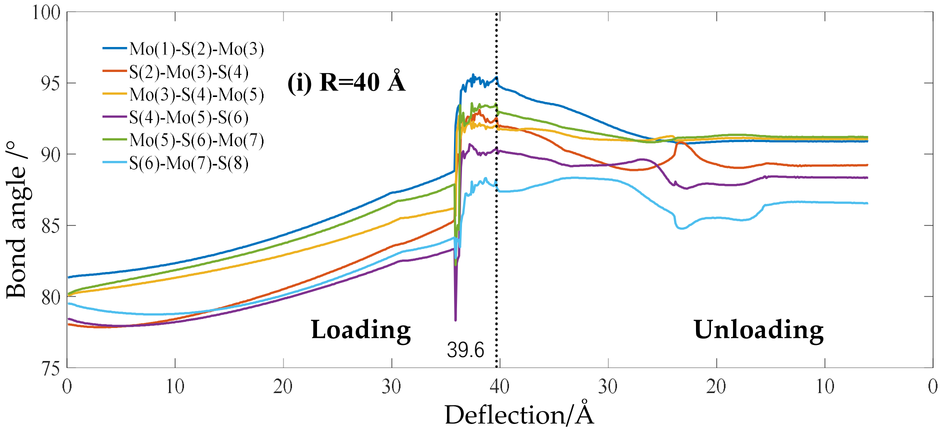
(i) R=40 Å
Mo(1)-S(2)-Mo(3)
S(2)-Mo(3)-S(4)
Mo(3)-S(4)-Mo(5)
S(4)-Mo(5)-S(6)
Mo(5)-S(6)-Mo(7)
S(6)-Mo(7)-S(8)
Bond angle /°
Deflection/Å
Loading
Unloading
39.6
100
95
90
85
80
75
0
10
20
30
40
30
20
10
0

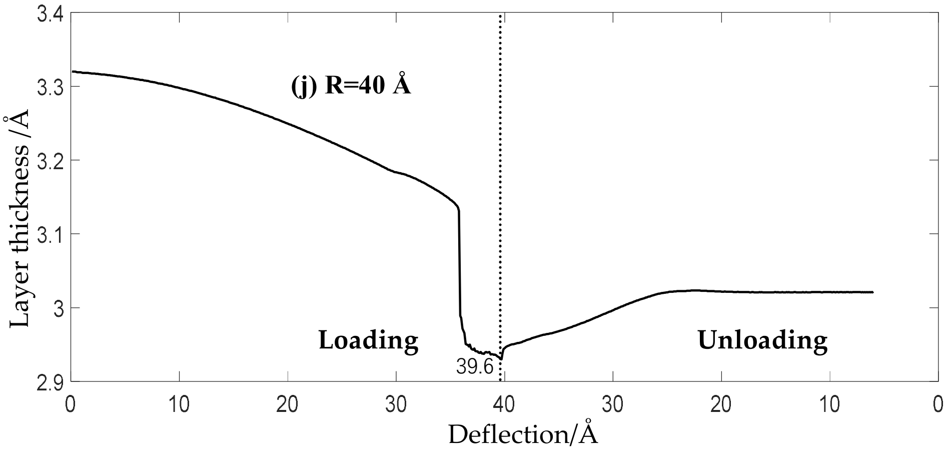
(j) R=40 Å
Layer thickness /Å
Deflection/Å
Loading
Unloading
39.6
3.4
3.3
3.2
3.1
3
2.9
0
10
20
30
40
30
20
10
0

Figure 1. The Mo-S and S-Mo bond lengths (a, d and h), S-Mo-S bond angles and Mo-S-Mo angles (b , e and i), and layer thickness of S-S (c, f and j) versus deflection during the loading process and unloading process with indenter radii of 15 Å, 20 Å and 40 Å (The labeled atoms are shown in Figure 3.).
